# Volumetric assessment of canines using post-mortem computed tomography (PMCT) for sexual dimorphism in a Malaysian population

**DOI:** 10.1186/s41935-023-00339-0

**Published:** 2023-03-17

**Authors:** Mohd Hafizal Harudin, Ademir Franco, Norhayati Jaffar, Mohamad Helmee Mohamad Noor, Mohamad Azaini Ibrahim, Scheila Manica

**Affiliations:** 1 Unit of Forensic Odontology, Department of Oral and Maxillofacial Surgery, Kuala Lumpur Hospital, Ministry of Health Malaysia, Kuala Lumpur, Malaysia; 2 Centre of Forensic and Legal Medicine and Dentistry, University of Dundee, Scotland, UK; 3 Unit of Forensic Radiology, Department of Radiology, Kuala Lumpur Hospital, Ministry of Health Malaysia, Kuala Lumpur, Malaysia; 4 Department of Forensic Medicine, National Forensic Institute of Malaysia (NFIM), Kuala Lumpur Hospital, Ministry of Health Malaysia, Kuala Lumpur, Malaysia

**Keywords:** Forensic dentistry, Sexual dimorphism, Sex estimation, Post-mortem computed tomography

## Abstract

**Background:**

Recent research has explored volumetric assessment in three-dimensional (3D) imaging procedures for sexual dimorphism. The 3D techniques have enabled a more realistic, accurate, and non-invasive visualization of sex-related anatomical parameters, such as the size and shape of human teeth.

**Aim:**

To perform sexual dimorphism based on dental tissue volumes of permanent left maxillary and mandibular canines in a Malaysian population.

**Methods:**

A cross-sectional study was carried out on 220 post-mortem computed tomography (PMCT) scans of Malaysian subjects (114 males and 106 females) aged between 18 and 40 years old. The permanent left maxillary and mandibular canines were analysed based on the volume of their (I) enamel cap, (II) coronal dentine, and (III) root, as well as the combination between the three dental tissue volumes (DTV). 3D Slicer version 4.10.2 computer software was used to perform a semi-automated segmentation of the anatomic regions of interest of each tooth.

**Results:**

The DTV of the permanent left maxillary and mandibular canines showed sexual dimorphism as the males presented larger DTV than females (*p* < 0.05). After binary logistic regression, the DTV revealed an overall sex classification rate of 60 to 74.1%. The DTV of the permanent left mandibular canine had more discriminant power to correctly classify males and females compared to the left maxillary canine.

**Conclusions:**

The volumetric assessment of the dental tissues of the permanent left maxillary and mandibular canines could discriminate sex in the Malaysian population. Given the limited classification rate, this approach could act solely as a supplementary tool to existing sex estimation methods.

## Background

Dental profiling relies on extracting biological evidence from teeth to estimate sex, age, ancestry, and other indicators to support human identification. In dental human identification, sex estimation is relevant to narrow lists of missing persons in mass disasters (Mânica et al. 2019). Recently, forensic specialists have developed various methods to distinguish males and females as part of the identification process (Manhaes-Caldas et al. 2019). The pelvic bones have been considered the most reliable indicator for sex estimation in skeletal remains, with accuracy rates above 95% (Luo 1995). Additionally, cranial and mandibular features demonstrate proper discriminant power as complementary tools (Williams and Rogers 2006; Hu et al. 2006). In practice, however, challenges may include bodies damaged by the fire, mutilated, or even skeletal remains with missing and broken bones (García-Campos et al. 2018). Given the resistance of teeth to environmental changes, they can be useful in these scenarios.

Previous studies suggested that sexual dimorphism could be assessed by odontometric analyses, specifically when it comes to measurements of the cervical mesiodistal and buccolingual diameters or via calculation of tooth-position indices (Khamis et al. 2014; Rao et al. 1989). In a recent systematic review, permanent mandibular canines figured as the most dimorphic teeth, followed by the permanent maxillary canines, molars, incisors, premolars, and mandibular incisors (Silva et al. 2019). However, the linear odontometric analysis of teeth led to inconsistent results so far and reduced the use of dental sexual dimorphism to an auxiliary tool in practice (Acharya and Mainali 2008).

In the contemporary forensic dental practice, imaging technology became more tangible. Three-dimensional (3D) evidence registration emerged, enabling experts to revisit the case in a digital scenario whenever necessary, using non-invasive features, and visualizing detailed anatomic structures. Similarly, odontometric dental analysis that were merely linear in the past are now volumetric (3D) (Bergmans et al. 2001). The application of computed tomography (CT) in forensic science is an example of imaging tools that brought reliable volumetric options for quantitative dental analyses (Kazzazi and Kranioti 2017). In previous studies using CT for the volumetric assessment of canines, the authors found significant differences between males and females (Manhaes-Caldas et al. 2019; García-Campos et al. 2018 Angelis et al. 2015; García-Campos et al. 2018). These studies were accomplished with samples from Europe, South America, and Africa. Since dental anatomy is also influenced by ancestry, population-specific investigations are justified to understand if the discriminant power of canine anatomy remains useful for sexual dimorphism when tested in different countries. No similar study was published on the Malaysian population.

Forensic odontology in Malaysia operates with special dedication to victims of natural mass disasters. These victims usually present degenerative cadaveric changes, such as decomposition, and require extensive and reliable post-mortem (PM) evidence registration. In this context, multi-slice PMCT imaging was introduced as part of the Malaysian medicolegal armamentarium. In parallel, dental profiling techniques, such as sexual dimorphism, were implemented to abbreviate the reconciliation phase (narrowing comparisons) and drive the identification process towards primary means for human identification, namely ridgeology, odontology, and genetics. Testing the proposed technology on a Malaysian population is a step forward to promote more evidence-based practice within Malaysia.

This study aimed to perform volumetric analyses of maxillary and mandibular canine anatomy for sexual dimorphism using PMCT scans of Malaysians. After the analyses, the study proposes the development of sex estimation formulae that isolate the segmented dental tissues and then combines them for volumetric quantification.

## Methods

### Sample

This study received approval from the Medical Research and Ethical Committee (MREC), Ministry of Health Malaysia (NMRR ID: NMRR-20–1817-55,718 (IIR)) on collecting post-mortem computed tomography images as study samples. The study was observational analytical and cross-sectional. The dental notation used in this study followed the World Dental Federation (*Fédération Dentaire Internationale*—FDI)*.*

An analytical observational (cross-sectional) study was performed. The sample consisted of 220 (PMCT) scans of subjects aged between 18 and 40 years old (114 males and 106 females), from a Malaysian population. A total 440 permanent left maxillary and mandibular canines (teeth #23 and #33) were assessed. The PMCT scans were retrospectively and randomly collected from an existing database. The images were obtained with a Toshiba Aquilion 64 CT unit (Toshiba, Minato, Japan) at the National Forensic Institute of Malaysia (NFIM), Kuala Lumpur Hospital. The inclusion criteria consisted of images of Malaysian males and females, aged between 18 and 40 years, that presented both the permanent left maxillary and mandibular canines in the same PMCT image. The exclusion criteria comprised decayed teeth #23 and #33, the presence of orthodontic appliances, restoration crowns, and periapical lesion in these teeth and images from individuals with unknown sex and date of birth. Images were obtained in *Digital Imaging and Communications in Medicine* (DICOM) format and were imported into the computer software 3D Slicer version 4.10.2 (The Slicer Community, International).

### Dental tissue visualization

**Fig. 1:**
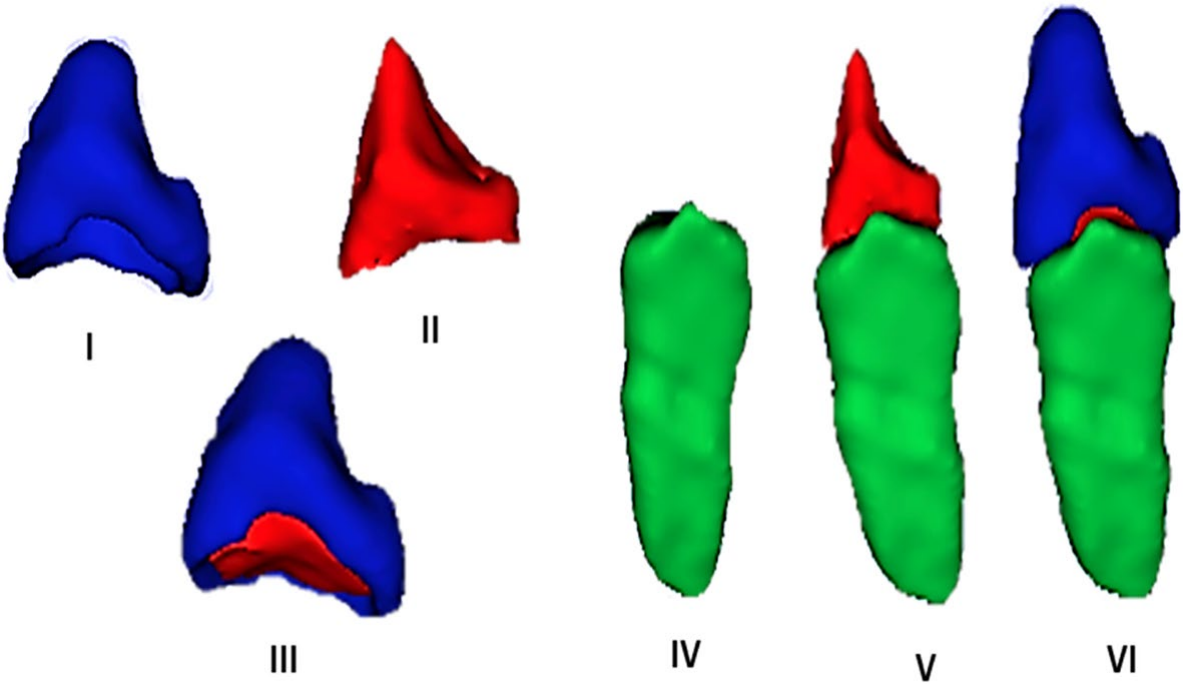
Six sets of dental tissue segmented and considered for sexual dimorphism in the present study. (I) Volume of enamel (Ve), (II) volume of coronal dentine and pulpal dentine (Vcdp), (III) total crown volume (VC), (IV) volume of the root (Vr), (V) total dentine including pulpal dentine (VTDP), and (VI) total tooth volume (VTT)

Dental tissue visualization was performed within the software following and adapting previous instructions (García-Campos et al. 2018). In the software, the selected PMCT images were initially enhanced based on contrast and brightness. Adjustments were made to distinguish the soft (dental pulp) and hard tissues (enamel, dentine, and alveolar bone). Magnification of up to 200% was used in the region of permanent left canines. Six sets of dental tissues were established for further quantification of volume (Fig. 1): (I) volume of enamel cap (Ve)—the enamel surface volume of the crown bounded by the outer enamel and the inner surface adjacent to the dentine junction; (II) volume of coronal dentin and pulpal dentin (Vcdp)—the volume of coronal dentin including coronal aspect of pulp chamber; (III) total crown volume (VC)—the total crown volume including enamel, coronal dentine, and pulpal dentine (Ve + Vcdp); (IV) volume of the root (Vr)—the volume enclosed by the basal crown surface of the root and the root apex; (V) total dentine, including pulpal dentine (VTDP)—the volume of total dentine including pulp (Vcdp + Vr); and (VI) total tooth volume (VTT)—represented by the volume of whole tooth (Vc + Vr).

Image segmentation was performed in multiplanar navigation, using axial, sagittal, and coronal views. In each view, the dental tissues were selected slice by slice and highlighted with a color system (blue for the enamel, red for the crown dentine, and green for the root). This process was performed by combining the *paint and level tracing* and *fill between slices* tool to select the tissues between slices semi-automatically. The cervical margin of the dental crown was defined in the axial plane using as reference the most apical extension of the enamel on the buccal or lingual/palatal surfaces on the sagittal reconstruction. In contrast, the tissue more apical to the reference was considered as root, and the remaining tissue was considered crown (Ma et al. 2013). Finally, the volume of each highlighted tissue was automatically calculated.

### Statistical analysis

Data analysis was performed using IBM SPSS 25.0 (IBM Corp., Armonk, NY, USA). Normality of distribution was tested using the Shapiro–Wilk analysis. Intraclass correlation coefficient (ICC) was performed to study intra- and inter-observer agreement (15% of the sample was reanalyzed 30 days after the main analysis by the main examiner [intra-observer] and an additional examiner [inter-observer]. Descriptive statistics of absolute and relative frequencies, and central tendency and dispersion, were calculated. Independent *t* test was used to analyze the sexual dimorphism, while binary logistic regression was applied to produce the sex estimation models. Accuracies based on dental tissue volume for sexual dimorphism was assessed separately for the permanent left maxillary and mandibular canines and the combination of both teeth. Statistical significance was set at 5% and the confidence interval at 95%.

## Results

The distribution of research data was normal, and intraclass correlation coefficient (ICC) for intra- and the inter-observer was 0.970 and 0.767, respectively.

Table 1 shows the descriptive data of dental tissues volumes of the permanent left maxillary and mandibular canines. The total number of PMCT imaging scans of each tooth (#23 and #33 are 114 and 106 for males and females, respectively). With both teeth combined, the total number of subjects was double. All dental tissues volumes sizes of tooth 23 and tooth 33 were found to be higher in males than in females. The same result was also found when combining the dental tissues volumes in both teeth. The independent *T* test showed a statistically significant difference between male and female size (*p* < 0.01) on each dental tissue of tooth 23 and tooth 33 and combination of both teeth.

**Table 1:** Descriptive analysis and Independent T-test for teeth 23 and 33 and for the combination of both

	**Dental Tissue**	**Male** ***N*** = 114		**Female** ***N*** = 106		**Independent ** ***T*** ** test**			**95% confidence interval of the difference**	
		**Mean**	**SD**	**Mean**	**SD**	***t***	**df**	**Sig**	**Lower**	**Upper**
**Tooth 23**	**Ve**	235.41	54.93	195.79	35.05	6.42	193.65	0.01	27.45	51.79
	**Vcdp**	130.57	39.08	113.32	27.80	3.80	204.36	0.01	8.23	26.22
	**VC**	366.00	84.47	309.12	55.45	5.94	196.58	0.01	37.99	75.74
	**Vr**	354.54	99.72	281.90	69.35	6.31	202.23	0.01	49.93	95.34
	**VTDP**	485.11	113.37	395.23	79.94	6.83	203.53	0.01	63.95	115.82
	**VTT**	720.52	146.17	591.02	98.87	7.74	199.56	0.01	96.09	162.91
**Tooth 33**	**Ve**	190.98	45.16	156.67	28.86	6.76	193.82	0.01	24.31	44.33
	**Vcdp**	124.34	40.50	101.45	29.50	4.76	218.00	0.01	13.41	32.36
	**VC**	315.32	77.73	258.12	53.49	6.40	201.24	0.01	39.57	74.84
	**Vr**	283.49	80.70	223.92	63.56	6.10	212.36	0.01	40.331	78.84
	**VTDP**	407.83	90.77	325.37	69.95	7.58	210.87	0.01	61.00	103.91
	**VTT**	598.81	112.34	482.04	87.14	8.65	211.33	0.01	90.15	143.40
		***N*** = 228		***N*** = 212						
**Tooth 23 + Tooth 33**	**Ve**	213.19	54.89	176.23	37.55	8.29	403.15	0.01	28.20	45.73
	**Vcdp**	127.46	39.83	107.39	29.21	6.06	415.87	0.01	13.55	26.58
	**VC**	340.65	84.88	283.62	60.06	8.18	409.55	0.01	43.33	70.94
	**Vr**	319.01	97.26	252.91	72.44	8.12	420.59	0.01	50.103	82.10
	**VTDP**	446.47	109.54	360.30	82.71	9.35	420.59	0.01	68.06	104.28
	**VTT**	659.66	143.66	536.53	107.83	10.21	419.66	0.01	99.44	146.83

**Note**: *N* number of samples, + combination, *SD* standard deviation, *t* computed test statistic, *df* degree of freedom, *Sig p* value, *Ve* volume of enamel, *Vcdp* volume of coronal dentine and pulp, *VC* total crown volume, *Vr* volume of root, *VTDP* total dentine and pulp volume, *VTT* total tooth volume

Table 2, Table 3, and Table 4 demonstrate the construction of equations with variables and constant values analyzed by means of binary logistic regression. Outcomes for the correct sex classification using the volume of the six dental tissues settings from teeth #23 and #33 are presented separately per tooth and combined. Each of the models for the six dental tissue settings showed significant difference at the 5% cut-point with a *p* value of 0.01. Odds ratios above 0.5 indicated females, while values below 0.5 indicated males. The overall percentage of correct sex predictions of dental tissue models for tooth #23 varied between 60 and 69.5%, while for tooth #33, prediction values varied from 67.7 to 74.1%. The combination of both teeth resulted in sex prediction values from 62.3 to 69.5%. The volume of the whole tooth (VTT) #33 reached the highest percentage for correct sex estimation, 74.1%, while the worst prediction was found using the volume of coronal dentine and pulpal dentine (Vcdp) of tooth #23 showed 60% individual and combined classification of females had minimum and maximum accuracy predictions ranging between 53 and 77%, respectively. In males, minimum and maximum accuracy predictions ranged between 62 and 71.9%, respectively. The logistic regression equations built for teeth #23 and teeth #33 are presented in the Appendix.

**Table 2:** Variables and constants used in the equations for sex prediction and their overall accuracy for tooth 23

Model	*B*	Wald	Sig	Exp (B)	95% C.I for EXP B		Prediction by sex (%)		Overall prediction (%)
					**Lower**	**Upper**	**Male**	**Female**	
**Ve**	− 0.021	28.292	0.01	0.979	0.972	0.987	70.2	67	68.6
Constant	4.363	-	-	-					
**Vcdp**	− 0.015	12.425	0.01	0.985	0.977	0.993	66.7	52.8	60
Constant	1.780	-	-	-					
**VC**	− 0.012	25.765	0.01	0.988	0.984	0.993	69.3	69.8	69.5
Constant	3.940	-	-	-					
**Vr**	− 0.010	28.747	0.01	0.990	0.987	0.994	64	66	65
Constant	3.036	-	-	-					
**VTDP**	− 0.010	31.642	0.01	0.991	0.987	0.994	64.9	63.2	64
Constant	4.090	-	-	-					
**VTT**	− 0.009	37.258	0.01	0.991	0.988	0.994	65.8	71.7	68.6
Constant	5.629	-	-	-					

**Note:** The odd cut value is 0.5, considering above this value is female and below is male, *B* coefficient for the constant, *Wald* Wald chi-square value, *Sig p* value, *Exp (B)* exponentiation of the *B* coefficient, *Ve* volume of enamel, *Vcdp* volume of coronal dentine and pulp, VC total crown volume, *Vr* volume of root, *VTDP* total dentine and pulp volume, *VTT* total tooth volume

**Table 3:** Variables and constants used in the equation for sex prediction and their overall accuracy for tooth 33

Model	*B*	Wald	Sig	Exp (B)	95% C.I for EXP B		Prediction by sex (%)		Overall prediction (%)
					**Lower**	**Upper**	**Male**	**Female**	
**Ve**	− 0.029	32.227	0.01	0.972	0.972	0.987	71.9	68.9	70.5
Constant	4.868	-	-	-					
**Vcdp**	− 0.020	18.677	0.01	0.980	0.972	0.989	65.8	67.9	67.7
Constant	2.134	-	-	-					
**VC**	− 0.014	29.427	0.01	0.986	0.981	0.991	67.5	67.9	67.7
Constant	3.940	-	-	-					
**Vr**	− 0.012	26.583	0.01	0.988	0.983	0.993	66.7	69.8	68.2
Constant	3.036	-	-	-					
**VTDP**	− 0.014	36.194	0.01	0.986	0.982	0.991	66.7	77.4	71.8
Constant	4.873	-	-	-					
**VTT**	− 0.013	42.931	0.01	0.987	0.983	0.991	71.9	76.4	74.1
Constant	5.629	-	-	-					

**Note:** The odd cut value is 0.5, considering above this value is female and below is male. *B* coefficient for the constant, *Wald* Wald chi-square value, *Sig* = *p* value, *Exp B* exponentiation of the *B* coefficient, *Ve* volume of enamel, *Vcdp* volume of coronal dentine and pulp, *VC* total crown volume, *Vr* volume of root), *VTDP* total dentine and pulp volume, *VTT* total tooth volume

**Table 4:** Variable and constants used in the equations for sex prediction and their overall accuracy for the combination of teeth 23 and 33

Model	*B*	Wald	Sig	Exp (B)	95% C.I for EXP B		Prediction by sex (%)		Overall prediction (%)
					**Lower**	**Upper**	**Male**	**Female**	
**Ve**	− 0.018	50.192	0.01	0.982	0.977	0.987	64	65.6	64.8
Constant	3.457	-	-	-					
**Vcdp**	− 0.017	30.547	0.01	0.983	0.977	0.989	65.4	59	62.3
Constant	1.919	-	-	-					
**VC**	− 0.011	49.887	0.01	0.989	0.986	0.992	67.1	62,7	65
Constant	3.395	-	-	-					
**Vr**	− 0.009	60.314	0.01	0.991	0.988	0.993	61.8	69.3	65.5
Constant	2.518	-	-	-					
**VTDP**	− 0.009	60.314	0.01	0.991	0.988	0.993	68	67.9	68
Constant	3.719	-	-	-					
**VTT**	− 0.008	67.903	0.01	0.992	0.990	0.994	70.6	68.4	69.5
Constant	4.731	-	-	-					

**Note:** The odds cut value is 0.5, considering above this value is female and below is male. *B* coefficient for the constant, *Wald* Wald chi-square value, *Sig* = *p* value, *Exp B* exponentiation of the *B* coefficient, *Ve* volume of enamel, *Vcdp* volume of coronal dentine and pulp, *VC* total crown volume, *Vr* volume of root, *VTDP* total dentine and pulp volume, *VTT* total tooth volume

## Discussion

The main factors involved in successful dental identification are the availability and completeness of ante-mortem dental records. In case of missing ante-mortem data, an alternative to the comparative analysis is dental profiling. In this process, forensic dentists investigate the teeth and the surrounding tissues of the oral cavity in order to estimate sex, age and ancestry by dental parameters (morphology or metric), habits, and nutritional deficiencies. Our research focused on sex estimation using volumetric tooth measurements. Dental sex estimation can be performed through the analysis of multiple teeth. More recently, for instance, the authors have found promising applications with the geometric morphometric analysis of 3D-scanned maxillary first premolars (Oliva et al. 2021). However, because of the consistency of permanent canines for sexual dimorphism previously demonstrated by the scientific literature (via computed tomography studies) (İşcan and Kedici 2003; Acharya and Mainali 2007; Tardivo et al. 2011; Tardivo et al. 2015), we based our analyses on these teeth.

The results of this study showed significant sexual dimorphism from the volumes of the permanent left maxillary and mandibular canines, either separately or in combination. The volume of dental tissues of males was higher than females. These results are consistent with the findings of Garcia-Campos et al. (2018), who tested similar parameters and methodology and suggested significant differences in dental tissue volumes of permanent maxillary and mandibular canines among the Sudanese population (except enamel volume of mandibular canines) (García-Campos et al. 2018 García-Campos et al. 2018). Similarly, studies carried out by Tardivo et al. (2011, 2015) and De Angelis et al. (2015) assessed the total tooth volume of canines in the European population and observed statistically significant sexual dimorphism, in which males presented higher volume values than females (Angelis et al. 2015; Tardivo et al. 2011; Tardivo et al. 2015). In terms of dental crown volume, Manhaes-Caldas et al. (2019) found statistically significant differences between sexes in Brazilian samples, with predominant volume values in males (Manhaes-Caldas et al. 2019). The larger canines in males could be attributed to genetic factors specified in sex-related genes (Schwartz and Dean 2005). The amelogenin genes on the X and Y chromosomes likely contribute to the proportion of dental tissues, with the X chromosomes being responsible for enamel thickness and the Y chromosomes possibly supporting dental growth (Schwartz and Dean 2005). Moreover, the addition of Y chromosomes to linked genes may justify an increase in mitotic activity in the dental lamina, which may contribute to differences in odontoblast activity between men and women (Harris et al. 2001).

According to the results of this study, the dental tissues of tooth #33 showed the highest overall accuracy for sexual dimorphism (74.1%) compared to dental tissues of tooth #23, and the combination of both. The obtained results agree with the study of Tardivo et al. (2015) that found better predictions from the total volume of mandibular canines compared to maxillary canines and corroborate the similar outcomes of Garcia-Campos et al. (2018); García-Campos et al. 2018; Tardivo et al. 2015). It must be noted, however, that a study on root volume by Kazzazi and Kranioti (2017) contradicts with our current results by claiming that the root of maxillary canines has better performance for sex estimation (Kazzazi and Kranioti 2017). Our study converges with most of the existing literature when it comes to canines. Specific forensic techniques for sexual dimorphism rely on these teeth, such as the canine index approach—with an overall accuracy of 72–74.8% (Reddy et al. 2008; Bakkannavar et al. 2015; Silva et al. 2016). This technique relies on tooth crown morphology and is metric instead of volumetric, but the outcomes remained similar to the present study—endorsing the dimorphic component on the development of tooth #33. When it comes to the Malaysian population, whole tooth crown morphology led to overall performance for sex predictions of 70.2–78.5% in previous investigations (Khamis et al. 2014).

To the best of our knowledge, this is the first study on tooth volume for sexual dimorphism in a sample of PMCT retrospectively collected from a Malaysian population. In forensic practice, sex of unknown individuals could be assessed by implementing the volumetric analysis of the whole mandibular canine. This approach could be considered evidence-based not only because of the present study but also because it is endorsed by a vast scientific literature. It must be noted, however, that sexual dimorphism with whole tooth volume should be an auxiliary tool in practice such as in dental profiling. Future studies in the field should consider population-specific testing of the present technique.

## Conclusions

PMCT scans enable the measurement of dental tissue volumes. This approach could be advantageous in improving dental profiling. Our study found a statistically significant difference between males and females using the volume of the whole permanent left mandibular canine. Additionally, males had larger teeth (#23 and 33) compared to females, with an overall sex estimation accuracy of up to 74.1%. The combination of volumes of maxillary and mandibular canines did not improve the accuracy for sex estimation.

## Data Availability

The data that support the findings of this study are available from the National Forensic Institute of Malaysia (NFIM), Department of Forensic Medicine, Kuala Lumpur General Hospital, Ministry of Health Malaysia, Malaysia, but restriction apply to the availability of these data, which were used under license for the current study, and so are not publicly available. Data are however available from the authors upon reasonable request and with permission of NFIM.

## Appendix

**Table:** Logistic regression equations for each dental tissues considering teeth #23 and #33 separately

Dental tissue	Tooth 23	Tooth 33
Ve	$P = \frac{{e}^{(4.363+(-0.021\times Ve)}}{1 + {e}^{(4.363+(-0.021\times Ve)}}$	$P = \frac{{e}^{(4.868+(-0.029\times Ve)}}{1 + {e}^{(4.868+(-0.029\times Ve)}}$
Vcdp	$P = \frac{{e}^{(1.78+(-0.015\times Vcdp)}}{1 + {e}^{(1.78+(-0.015\times Vcdp)}}$	$P = \frac{{e}^{(2.134+(-0.020\times Vcdp)}}{1 + {e}^{(2.134+(-0.020\times Vcdp)}}$
VC	$P = \frac{{e}^{(3.94+(-0.012\times VC)}}{1 + {e}^{(3.94+(-0.012\times VC)}}$	$P = \frac{{e}^{(3.94+(-0.014\times VC)}}{1 + {e}^{(3.94+(-0.014\times VC)}}$
Vr	$P = \frac{{e}^{(3.036+(-0.01\times Vr)}}{1 + {e}^{(3.036+(-0.01\times Vr)}}$	$P = \frac{{e}^{(3.036+(-0.012\times Vr)}}{1 + {e}^{(3.036+(-0.012\times Vr)}}$
VTDP	$P = \frac{{e}^{(4.090+(-0.021\times VTDP)}}{1 + {e}^{(4.090+(-0.021\times VTDP)}}$	$P = \frac{{e}^{(4.873+(-0.014\times VTDP)}}{1 + {e}^{(4.873+(-0.014\times VTDP)}}$
VTT	$P = \frac{{e}^{(5.629+(-0.009\times VTT)}}{1 + {e}^{(5.629+(-0.009\times VTT)}}$	$P = \frac{{e}^{(5.629+(-0.0013\times VTT)}}{1 + {e}^{(5.629+(-0.0013\times VTT)}}$

The odd cut value is 0.5, considering above this value is female and below is male

*
Ve* Volume of enamel, *Vcdp* Volume of coronal dentine and pulp, *VC* Total crown volume, *Vr* Volume of root, *VTDP* Total dentine and pulp volume, *VTT* Total tooth volume, *FDI* World Dental Federal notation (#23 and 33)

## Abbreviations

2D: Two dimensional
3D: Three dimensional
AM: Ante-mortem
AMEL: Amelogenin
BL: Buccolingual
CT: Computed Tomography
COVID-19: Coronavirus disease 2019
df: Degree of freedom
DICOM: Digital Imaging and Communications in Medicine
DNA: Deoxyribonucleic acid
ICC: Intraclass correlation coefficient
MD: Mesiodistal
MREC: Medical Research and Ethical Committee
MRI: Magnetic resonance imaging
NFIM: National Forensic Institute Malaysia
OR: Odds of ratio
PCR: Polymerase chain reaction
PM: Post-mortem
PMCT: Post-mortem computed tomography
SPSS: Statistical Package for the Social Sciences
VC: Total volume of crown
Vcdp: Volume of coronal dentine and pulp
Ve: Volume of enamel
Vr: Volume of root
VTDP: Total volume of dentine and pulp
VTT: Total tooth volume
